# Development and initial validation of a reliable German self‐report measure to assess acute cannabis intoxication‐effects (CanTox‐17)

**DOI:** 10.1002/mpr.1925

**Published:** 2022-06-16

**Authors:** Merle Schüler, Steffen Moritz, Thomas Schnell

**Affiliations:** ^1^ Medical School Hamburg University of Applied Sciences and Medical University Hamburg Germany; ^2^ Department of Psychiatry and Psychotherapy University Medical Center Hamburg‐Eppendorf Hamburg Germany

**Keywords:** cannabis, intoxication, psychosis, questionnaire, substance use disorder

## Abstract

**Objectives:**

Both positive and negative including psychotic‐like cannabis intoxication effects are well‐established. Yet, consequences for consumption patterns, addictive behavior or psychotic developments are poorly researched in general, in Germany not at all. Thus, objective was to develop the first German‐language questionnaire operationalizing acute cannabis intoxication effects, based on the original “Cannabis Experience Questionnaire” (CEQ).

**Methods:**

After expert translation of the CEQ, items related to acute intoxication effects were presented to a sample of 537 cannabis users. Four‐ and five‐factorial solutions of explorative factor analysis with a randomly split sub‐sample 1 were cross‐validated by confirmatory factor analysis on sub‐sample 2.

**Results:**

After content review of factors and analysis of external validity, a 17 item four‐factorial model was approved. Factors are (1) “paranoia/dysphoria”, (2) “confusion/disorientation”, (3) “euphoria/creativity”, (4) “psychosis‐like/loss of reality”. Model fit is satisfactory (RMSEA = 0.058, SRMR = 0.065, CFI = 0.929, TLI = 0.914). Correlations with corresponding external measures support construct validity.

**Conclusions:**

The present questionnaire is a time‐efficient, valid and reliable instrument. Thus, predictors of different cannabis intoxication effects can be analysed for the first time in German‐speaking area, as well as their significance for substance use or psychosis risk.

## 1 INTRODUCTION

From a continuum perspective, psychotic experiences range from schizophrenia to psychotic‐like experiences (PLE) in the general population without underlying psychiatric disorder (Cowan & Mittal, 2020; Lee et al., 2016). Between 2% and 16% of the general population report lifetime PLE, such as depersonalization, delusions and hallucinations, usually transient and/or attenuated in nature (van Os, Myin‐Germeys, Delespaul, & Krabbendam, 2009). They are associated with increased risk for psychosis (Cowan & Mittal, 2020) and therefore may reflect increased vulnerability (Unterrassner et al., 2017). A critical period for the occurrence of PLE as well as the manifestation of psychotic disorders is young adulthood (Mustonen et al., 2018; Ragazzi et al., 2018) and thus the age at which cannabis use is particularly frequent (Orth & Merkel, 2019).

Cannabis users report different acute and subacute intoxication experiences, ranging from relaxation to stimulation, euphoria to anxiety and, in higher doses, hallucinations (Baggio et al., 2014). If acute effects resemble psychotic symptoms, they are described as cannabis‐induced psychosis‐like experiences (PLE) (Schmid et al., 2020). Interaction effects between neurobiological vulnerability to psychosis and cannabinoids or the endogenous cannabinoid system (ECS) were assumed (Hamilton, 2017). Cannabis use is therefore regarded a risk factor for both psychosis (Gage et al., 2016; Sideli, Quigley et al., 2020) and PLE (Fonseca‐Pedrero et al., 2020; Karcher et al., 2019). Concurrent evidence was provided by Quinn et al. (2017), who identified correlations between paranoid‐dysphoric cannabis intoxication and PLE in everyday life. Similarly, paranoid‐dysphoric cannabis effects were associated with schizotypal personality traits, considered as risk or vulnerability markers of psychosis (Takahashi et al., 2021). As the onset of cannabis use typically occurs in early adolescence (Orth & Merkel, 2019), the age at which prodromal psychotic developments run their course, cannabis‐induced PLE may be of interest for early psychosis detection and intervention.

Based on preliminary estimates, users with cannabis‐induced PLE may have an up to fivefold increased risk of psychosis compared to PLE‐naive users (McHugh et al., 2017). The former were also younger at their first use and used cannabis more frequently. Interestingly, users without psychosis‐like acute effects had a similar risk of psychosis compared to cannabis‐naive individuals (McHugh et al., 2017).

Vulnerability to psychosis seems to be fundamentally associated with an increased sensitivity to cannabinoids. Both first‐episode psychosis and schizotypy patients appeared to be more sensitive to pleasurable as well as psychosis‐like cannabis effects compared with controls (Bianconi et al., 2016; Stirling et al., 2008). However, Sami et al. (2019) just found increased occurrence of psychosis‐like but not euphoric intoxication effects in first episode psychoses. Abnormalities in the ECS may correspond with both sensitivity to cannabinoids and vulnerability for psychosis (Giuffrida et al., 2004). Independent of cannabis use, patients with schizophrenia have increased cannabinoid receptor density and elevated levels of endocannabinoids, and Weiser and Noy (2005) concluded that increased vulnerability is associated with increased risk of using cannabis even before initial psychotic manifestation. In particular, early onset and intensive use could be understood as first prodromal signs of psychotic development (Ksir & Hart, 2016). And possibly, cannabis‐induced PLE could be further signs of a psychopathological process.

### 1.1 Cannabis intoxication effects and cannabis discontinuation

Interaction effects between cannabinoids and vulnerability for psychosis may suggest increasing incidences of schizophrenia, as cannabis use has increased in recent decades (Orth & Merkel, 2019). In the subgroup of “early‐onset” psychoses, for example, comorbid cannabis use is present in 30%–40% (Moulin et al., 2018; Sami & Bhattacharyya, 2018). Continued cannabis use in manifest psychotic disorders is also associated with various negative consequences such as increased relapse rates and more severe symptoms (Hasan et al., 2020). However, epidemiological data indicate consistent incidences of psychotic disorders. As a relativizing influencing factor, it is discussed whether the nowadays high‐potency cannabis leads to a discontinuation of use (discontinuation hypothesis) in some schizophrenia risk persons with high sensitivity to cannabinoids due to aversive psychoactive effects (Sami et al., 2019). Increased cessation of cannabis use could put a potential increase in the incidence of schizophrenia into perspective. Schoeler et al. (2016) also confirmed that first‐manifest patients who quit use subsequently had fewer psychotic relapses, fewer inpatient admissions, and shortened treatment times compared with patients who continued cannabis use. Accordingly, Sami et al. (2019) report that abusers are more likely to quit consumption and exhibit higher abstinence motivation when they have experienced PLE as an intoxication effect. Pleasant acute effects, on the other hand, were associated with continued use and low abstinence motivation, which also touches on the issue of substance use disorder in the medium to long term (Bianconi et al., 2016). Especially in the initial phase of use, acute effects seem to be predictively relevant, since early positive but not negative effects of use are associated with later dependence (Fergusson et al., 2003; Le Strat et al., 2009). In later stages of use, acute effects appear to be less operant. Here, the anticipation of the earlier positive effects and, at the same time, the momentum of addiction are likely to be of greater behavioral relevance.

### 1.2 Operationalization of acute intoxication effects

Barkus et al. (2006) developed a 56 item “Cannabis Experiences Questionnaire” (CEQ) to operationalize subjective experiences to cannabis use both during and after intoxication. An initial exploratory factor analysis (EFA) suggested a 3‐factor solution (Stirling et al., 2008) with “psychotic‐dysphoric” after‐effects, “expansive” after‐effects and an “intoxication index”. Conducting a second EFA, a 4‐component solution better explained data (Barkus & Lewis, 2008). Acute intoxication effects were represented by two factors, “paranoid‐dysphoric experiences” and “euphoric experiences”. After‐effects were represented by two further factors, “amotivational after‐effects” and “psychosis like after‐effects”. In Birnbaum et al. (2017), the “after‐effect” items of the instrument were difficult to separate distinctly from acute intoxication effects, but were dispersed across all subscales rather than comprising a distinct “after‐effects” subscale. Finally, Quinn et al. (2017) did not even attempt to differentiate acute from after‐effects and analysed only those 43 items that focus on acute intoxication effects within a student sample. They suggested two distinct “euphoric” and “paranoid‐dysphoric experiences” factors.

### 1.3 Aim of the present study

A German‐language instrument to operationalize subjective acute cannabis experiences does not exist, so that German‐language research has not yet addressed predictors and implications of differential cannabis effects. The development and evaluation of a corresponding instrument was the aim of the present investigation.

## 2 METHODS

### 2.1 Inclusion criteria and study procedures

First, the items for a German‐language version of the CEQ were developed (see Section 2.2) and a questionnaire battery was prepared for an online survey. This was followed by the recruitment of German‐speaking subjects between 18 and 35 years of age who had used cannabis within the last three months. Diagnosis of mental disorders, especially psychosis, and/or psychiatric treatment (current or history) were exclusion criteria, as well as the use of other psychoactive substances except cannabis. After inclusion criteria were requested and written informed consent was obtained, subjects were directed to answer the online questionnaire. Recruitment took place between December 2019 and June 2020 on social media (Facebook, Reddit, cannabis forums). All procedures were performed in accordance with the latest revision of the Declaration of Helsinki.

### 2.2 Construction of a German‐language “Cannabis Experience Questionnaire” (CEQ)

56 items of the original version of the CEQ by Barkus et al. (2006) were translated into German according to the criteria of Brislin (1970): Frist, a bilingual student translated the items from English into German. Second, a back translation was done by another bilingual student. Both versions were compared for equivalence, discrepancies were discussed and revised.

Translation was followed by an expert validation of the items. For this purpose, the items were inspected by two psychiatric experts and two persons who have been using cannabis regularly for many years and who are well acquainted with the corresponding scene. They rated each item in terms of linguistic appropriateness and content validity. Both aspects were measured using a 4‐point Likert scale, with dimensions ranging from (1) “unclearly expressed” or “inappropriate” to (4) “clearly expressed” or “appropriate.” Items coded 4 were retained, and items coded 3 were revised. Codings of 1 or 2 resulted in exclusion of the item.

The experts criticised what Birnbaum et al. (2017) also reported after his evaluation of the original CEQ version, namely difficulties with the discriminatory power of items that should differ between acute intoxication effects and after‐effects of cannabis use. Items related to acute intoxication should actually represent a different period of intoxication compared with the “after‐items” related to subacute intoxication. But in Birnbaum et al. (2017), acute‐effect items and after‐effect items could not be assigned to different factors according to their content, but they were mixed within factors. Our experts' feedback after validation of the items corresponded in content to the factor analytic phenomenon in Birnbaum's study. The two intoxication states acute versus subacute seemed difficult to distinguish. For this reason and in line with the study of Quinn et al. (2017), it was decided to focus exclusively on items that clearly depict the acute intoxication state, each quantified by a five‐point Likert scale, according to the UK‐ template from “never” to “almost always”.

### 2.3 Further survey instruments

#### 2.3.1 Sociodemographic data, cannabis use patterns and mental health

Information on sociodemographic parameters included age, gender, education level, and occupation. This was followed by questions on cannabis use, that is, age at onset of use and average frequency of use (joints per week). Regarding mental health, participants were asked to indicate whether they had ever been diagnosed with a mental disorder and whether they had ever been in outpatient or inpatient psychiatric treatment. In order to get an approximate impression of family vulnerabilities, the respondents were also asked whether there were any known mental disorders in the family. If known, corresponding diagnoses should be given.

#### 2.3.2 Validation measure: Community psychic experiences (CAPE)

For external validity, the German‐language version of the CAPE (Schlier et al., 2015) was used, consisting of 42 items, each coded on a four‐point rating scale (never, sometimes, often, almost always). The evaluation forms a CAPE total score as well as three subscales depicting psychotic experience in the general population (positive symptoms, negative symptoms depressive symptoms). The German‐language CAPE achieves good internal consistency (Cronbach's alpha = 0.81–0.85) and has been successfully tested for external validity using various measures (Schlier et al., 2015).

### 2.4 Statistics

#### 2.4.1 Random split of the sample and exploratory factor analysis (EFA)

To reduce the risk of random or extragenic variables affecting model fit, random split was used to divide the sample into two subsamples so that two separate samples could be imitated.

Sample adequacy and factorability of data were analysed using measure of sampling adequacy (MSA), the KMO‐measure (Kaiser‐Meyer‐Olkin) and the Bartlett Test for sphericity. Parallel analysis, scree‐test, and MAP‐test were used to estimate the number of factors to be extracted. The content interpretability of the factors was also considered to select the number of factors. Exploratory factor analysis using the maximum likelihood method with Varimax rotation and Kaiser normalization was performed on the data from subsample 1 to capture the underlying factor structure. Items with main loadings <0.40 and with ambiguous loadings with a difference between main and secondary loading(s) < 1 were excluded. Also, a quasi‐exploratory optimization of the identified model structure was performed by including modification indices, and items with a corrected discriminant power <0.30 were excluded (Field, 2018). Procedures were performed using IBM Statistical Package for Social Sciences (SPSS) version 26 and R Studio (package: psych).

#### 2.4.2 Cross‐validation by confirmatory factor analysis (CFA)

Confirmatory factor analysis was performed on subsample 2 to validate the final exploratory and quasi‐exploratory optimized factor structure using R Studio (packages: haven, lavaan) (so‐called cross‐validation). Since the χ^2^‐test is considered to be sample sensitive, the ratio between χ^2^‐and the degrees of freedom was additionally formed (χ^2^/*df*) (Schmermelleh‐Engel et al., 2003). A good fit is considered to be 0 = χ^2^/df ≤ 2, and an acceptable fit is 2 < χ^2^/*df* ≤ 3. In addition, the root mean square error of approximation (RMSEA) and the standardized root mean square residual (SRMR), the comparative fit index (CFI), and the Tucker Lewis index (TLI) should be used (Schmermelleh‐Engel et al., 2003). In accord with Browne and Cudeck's (1993) and Vandenberg and Lance (2000) criterion, CFI and TLI >0.90 and RMSEA and SRMR <0.08 indicated satisfactory model fit.

#### 2.4.3 Reliability analysis

The reliability of the final factor and item structure was tested using internal consistency (Cronbach's alpha). In addition, the item analysis included the difficulty indices, the corrected item discrimination value and the alpha coefficient of the items. Regarding internal consistency, Cronbach's alpha with values around 0.8 are recommended. The preferable range for item difficulty is 0.2–0.8, and good corrected item discrimination indicate indices ˃ 0.5 (Nunnally & Bernstein, 1994). Items with discriminatory power below 0.3 should be rejected.

#### 2.4.4 Analyses of validity

As a measure to examine the validity of the instrument, the individual factors were correlated with the higher‐level as well as with the subscales of the CAPE for external validity. In addition, the extent to which the explored factors represent distinct constructs was examined. According to Cohen et al. (2003), the intercorrelation of the factors should be below *r* = 0.70.

## 3 RESULTS

### 3.1 Sample characteristics

Of initial 600 recruited individuals, 63 did not meet with inclusion criteria or did not finish the questionnaire. The final sample consisted of 537 participants. The sample was thus large enough for a random split (subsample 1, *n* = 268; subsample 2, *n* = 269). Subsamples were analysed for differences with regard to sociodemographics, patterns of cannabis use and clinical parameters using independent‐samples *t*‐tests and Mann‐Whitney *U* test, the latter if requirements for a *t*‐test were not met. There were no significant differences between subsamples (Table 1).

**TABLE 1:** Comparison of subsamples in terms of baseline data collected

	Descriptive data		Statistics	
	EFA, Sample 1 (*n* = 268)	CFA, Sample 2 (*n* = 269)	Value	*p*
Gender (m/w/d)	168/96/4^4^	168/100/1^4^	−0.045^2^	0.964
Age (Years)	23.78 (3.99)^1^	23.90 (3.67)^1^	−0.365^3^	0.716
Education^5^	9/14/21/119/33/72^4^	8/15/24/115/47/60^4^	−0.415^2^	0.678
Marital status^6^	130/124/10/4^4^	138/119/11/1	−0.700^2^	0.484
Age at onset of cannabis use	16.65 (2.59)^1^	16.91 (3.07)^1^	−1.010^3^	0.313
Joints per week (within last month)	10.22 (15.82)^1^	10.01 (17.20)^1^	−0.611^2^	0.541
Diagnose of mental disorder	59 (22.0%)^4^	63 (23.4%)^4^	−0.365^2^	0.715
Outpatient psychotherapy	52 (19.4%)^4^	51 (19.0%)^4^	−0.131^2^	0.896
Inpatient treatment	15 (5.6%)^4^	19 (7.1%)^4^	−0.572^2^	0.567

*Note*: ^1^, M (SD) mean (standard deviation); ^2^, Mann‐Whitney *U* test; ^3^, *t* test; ^4^, frequencies (percentages in parentheses); ^5^, secondary school (8 years), secondary school (10 years), professional baccalaureate, baccalaureate, vocational training, university degree; ^6^, single, steady partnership, married, divorced; *p*, significance level.

### 3.2 Dealing with missing values

Analysis of missing values revealed a proportion of missing values of 0.71% (minimum value = 0.2%; maximum value = 2.6%) at the case and variable levels. To analyse random occurrence of missing values, Little's missing completely at random test was performed. Neither the values of the new developed questionnaire (*χ* ^2^ = 392.410, *df* = 415, *p* = 0.781) nor the CAPE (*χ* ^2^ = 515.982, *df* = 587, *p* = 0.984) revealed significance as predicted. Consequently, missing values could be imputed using the expectation‐maximization method.

### 3.3 Exploratory factor analysis (subsample 1, *n* = 268)

To determine the factor structure, all items underwent an EFA with data of sub‐sample 1 (*n* = 268). The Kaiser‐Meyer‐Olkin coefficient (KMO) was 0.867 and implied a good fit of the correlation matrix for factor analysis. Bartlett's test was significant as required (*p* < 0.001). All items reached the required MSA value of > 0.60 (Bühl, 2018). The significance level was *p* < 0.001. Regarding the extraction of factors, Parallel analysis and Scree‐Test indicated a four to six‐factor solution. Kaiser's criterion indicated a six factor solution. Since the six‐factor solution included a factor with only two items and was difficult to interpret in terms of content, a four‐factor and a five‐factor solution were chosen, both optimized to their final factor structures. To reduce the models, all items with a principal loading below 0.40 were first removed. In a second reduction step, items with a difference of main loading to secondary loadings <1 were removed. The remaining factors were quasi‐exploratory optimized using the modification indices.

The five‐factor solution integrated 19 items and explained a total variance of 51.2%; the four‐factor solution integrated 17 items and explained a total variance of 59.5%. Table 2 presents the items and indicates their respective assignment to the factors of the two solutions. Table 3 presents the two factor solutions with factor loadings.

**TABLE 2:** Items of the four‐ and five‐factor solutions

Nr.	Content	4 Factors	5 Factors
2	How often did you feel fearful while smoking marijuana?	x	Factor 1
4	How often did you feel paranoid while smoking marijuana?	Factor 1	
6	How often did you feel anxious while smoking marijuana?		
13	How often did you feel nervous while smoking marijuana?		
22	How often did you feel depressed while smoking marijuana?		x
14	How often did you have your speech become slurred while smoking marijuana?	Factor 2	Factor 2
15	How often did you have the sensation that time had slowed down while smoking marijuana?		
28	How often did you feel disturbed in your thinking while smoking marijuana?		
38	How often did you feel like you weren't fully aware of what was going on while smoking marijuana?		
18	How often did you feel like you were able to understand the world better while smoking marijuana?	Factor 3	Factor 4
31	How often did you feel energized while smoking marijuana?		x
33	How often did you feel full of ideas while smoking marijuana		Factor 4
34	How often did you feel more creative while smoking marijuana?		
10	How often did you feel threatened by an unknown force while smoking marijuana?	Factor 4	Factor 3
16	How often did you hear voices when there was no one there while smoking marijuana?		x
19	How often did you feel like you lost your sense of reality while smoking marijuana?		x
21	How often were you afraid that you were going crazy/mad while smoking marijuana?		x
29	How often did you feel like you no longer know yourself while smoking marijuana?		Factor 3
25	How often did you feel relaxed while smoking marijuana?	x	Factor 5
30	How often did you feel sleepy while smoking marijuana?	x	
1	How often did you feel happy while smoking marijuana?	x	
35	How often did you feel angry while smoking marijuana?	x	Factor 3
5	How often did you feel uncomfortably sleepy while smoking marijuana?	x	
7	How often did you feel like there was something which you had to do no matter what, or feel compulsive while smoking marijuana?	x	

*Note*: Labels for the factors of the four‐factor solution: Factor 1 = paranoia/dysphoria; factor 2 = confusion/disorientation; factor 3 = euphoria/creativity; factor 4 = psychosis‐like/loss of reality.

Labels for the factors of the five‐factor solution: Factor 1 = anxiety; factor 2 = confusion/disorientation; factor 3 = psychosis‐like (CAVE: problematic content assignment of items); factor 4 = euphoria/creativity; factor 5 = contentment and serenity.

Evaluation was done on the items translated into German. The 17‐item version German questionnaire will be made available on request.

**TABLE 3:** Factor loadings of the four‐ and five‐factor solutions

	4‐Factors				5‐Factors				
Item	Factor 1	Factor 2	Factor 3	Factor 4	Factor 1	Factor 2	Factor 3	Factor 4	Factor 5
2					0.81				
4	0.67				0.62				
6	0.81				0.60				
13	0.74				0.61				
22	0.66								
14		0.69				0.60			
15		0.73				0.65			
28		0.74				0.65			
38		0.75				0.65			
18			0.70					0.55	
31			0.65						
33			0.86					0.81	
34			0.86					0.86	
10				0.72			0.57		
16				0.77					
19				0.55					
21				0.57					
29				0.53			0.61		
25									0.84
30									0.73
1									0.49
35							0.75		
5							0.46		
7							0.41		

*Note*: Labels for the factors of the four‐factor solution: Factor 1 = paranoia/dysphoria; factor 2 = confusion/disorientation; factor 3 = euphoria/creativity; factor 4 = psychosis‐like/loss of reality.

Labels for the factors of the five‐factor solution: Factor 1 = anxiety; factor 2 = confusion/disorientation; factor 3 = psychosis‐like (CAVE: problematic content assignment of items); factor 4 = euphoria/creativity; factor 5 = contentment and composure.

### 3.4 Confirmatory factor analysis (subsample 2, *n* = 269)

Confirmatory factor analysis was conducted on the second subsample to verify the explored factor structure. Both the four‐factor solution and the five‐factor solution achieved good model fits. The goodness‐of‐fit indices of the four‐ and five‐factor solutions displays Table 4.

**TABLE 4:** Fit indices of the factor solutions in comparison

Model	χ^2^	df	χ^2^/df	RMSEA	SRMR	CFI	TLI
4 Factors	215.994*	113	1.91	0.058	0.065	0.929	0.914
5 Factors	244.221*	142	1.72	0.054	0.062	0.943	0.932

*Note*: *χ^2^ is significant at the level of *p* < 0.001.

CAVE: In spite of the better scores of the five factors, the final decision was made in favor of the four factors, due to content‐related aspects as well as external validity scores.

#### 3.4.1 Content analysis of the factors and decision for the four‐factor solution

The content inspection of the different factors led to their following designations:

Factors of four‐factor solution: factor 1 = paranoia / dysphoria; factor 2 = confusion / disorientation; factor 3 = euphoria / creativity; factor 4 = psychosis‐like / loss of reality.

Factors of the five‐factorial solution: Factor 1 = anxiety; factor 2 = confusion/disorientation; factor 3 = psychosis‐like (CAVE: problematic content assignment of some items); factor 4 = euphoria/creativity; factor 5 = contentment and composure.

The final decision in favor of the four‐factor solution and against the five‐factor solution was based firstly on the inspection of the content of the factors. In particular, factor 3 of the five‐factor solution appeared difficult to interpret in terms of content, since two items (sleepiness and compulsivity) did not fit well with the other psychosis‐like item contents. Also, the external validity of this factor was low, as indicated by the low correlation with the corresponding CAPE scale (see below). Conversely, the four‐factorial solution showed a very high fit with the CAPE scales (see Section 3.6.2). Finally, the five‐factorial solution displayed a lower variance explanation than the four factors, and the difficulty indices of factor 5 are all in a questionably high range (values around 0.8, see below). The 17 items of the four‐factor solution thus form a new measurement instrument, which was named CanTox‐17 (abbreviation for cannabis intoxication).

**FIGURE 1:**
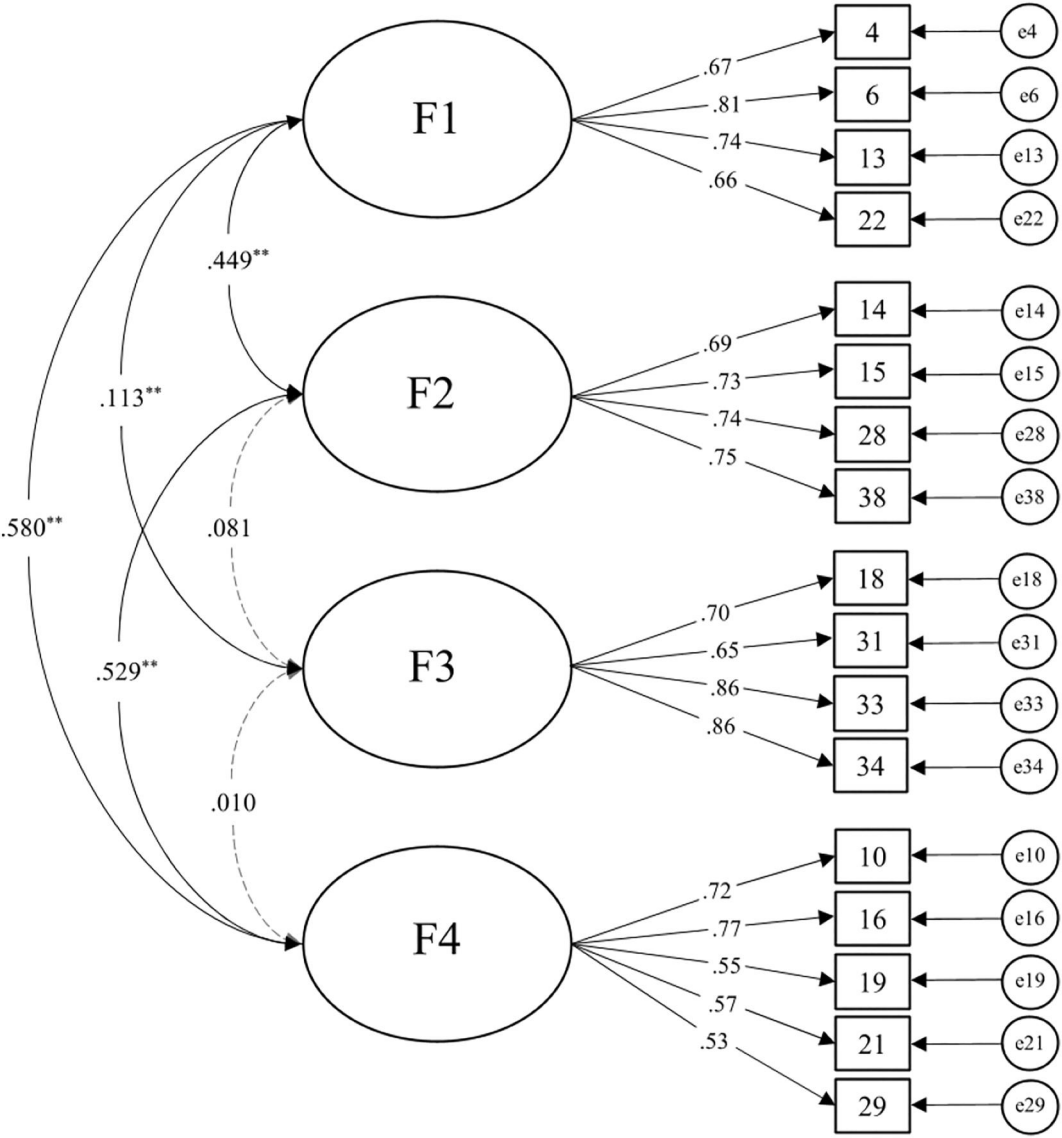
Path model. Note. Dashed lines = Factor‐correlations < 0.10; Factor 1 = paranoia/dysphoria; Factor 2 = confusion/disorientation; Factor 3 = euphoria/creativity; Factor 4 = psychosis‐like/loss of reality

The path model of the final four‐factorial structure presents Figure 1.

### 3.5 Item and scale analysis (reliability analysis) of the four factor solution

Inspection of descriptive data reveale that pleasurable cannabis experiences exceeded negative experiences within our sample. Psychotic‐like experiences seem to occur least frequently (see Table 5 for means and standard deviations).

**TABLE 5:** Reliability and item analysis of the final factor structure (four‐factor solution)

Factor	*m (SD)*	Item Nr.	Difficulty	Corrected discrimination	Alpha coefficient
1 paranoia/dysphoria	2.06 (0.93)	4	0.412	0.567	0.735
	2.14 (0.94)	6	0.420	0.633	0.699
	2.09 (0.92)	13	0.430	0.604	0.720
	1.87 (0.79)	22	0.427	0.558	0.750
**Factor 1 total**	**2.04** (**0.89**)				**0.772** ^**1**^
2 confusion/disorientation	2.23 (1.05)	14	0.450	0.527	0.726
	3.28 (1.16)	15	0.665	0.537	0.724
	2.97 (1.09)	28	0.595	0.588	0.694
	2.23 (1.01)	38	0.450	0.606	0.687
**Factor 2 total**	**2.67** (**1.07**)				**0.764** ^**1**^
3 euphoria/creativity	2.77 (1.19)	18	0.553	0.514	0.769
	3.31 (0.98)	31	0.502	0.433	0.796
	3.12 (1.04)	33	0.632	0.727	0.653
	3.26 (1.09)	34	0.652	0.690	0.669
**Factor 3 total**	**3.12** (**1.08**)				**0.780** ^**1**^
4 psychosis‐like/loss of reality	1.17 (0.49)	10	0.233	0.522	0.704
	1.16 (0.47)	16	0.231	0.422	0.728
	1.66 (0.89)	19	0.301	0.573	0.681
	1.46 (0.80)	21	0.299	0.556	0.674
	1.42 (0.78)	29	0.284	0.553	0.676
**Factor 4 total**	**1.38** (**0.69**)				**0.740** ^**1**^

*Note*: *n* = 537; ^1^, Cronbach's Alpha; m (SD), mean (standard deviation). Bolded values indicate the clarity of exposition.

To determine the internal consistency of the four factors, Cronbach's alpha was determined. Values between *α* = 0.74 and *α* = 0.78 represent good internal consistency. As part of the item analysis, the corrected item discriminant power, difficulty indices, and alpha coefficient were determined. All items displayed values within an acceptable range of values. The corrected item discriminants range from 0.422 to 0.727. The difficulty indices range from 0.23 to 0.66.

Table 5 presents the characteristic values of the item and scale analysis.

### 3.6 Intercorrelations of scales and external validity of CanTox‐17

#### 3.6.1 Scale intercorrelations

The three scales of the CanTox‐17 representing negative acute effects correlate significantly at a level between *r* = 0.44 ‐ 0.58 with each other (factors 1, 2 and 4). The factor representing positive acute effects (factor 3) does not correlate significantly with factors 2 and 4, and has a very low significant correlation of *r* = 0.113 with factor 1. Since all values are below *r* = 0.70, the four factors are considered sufficiently independent of each other by definition (Cohen et al., 2003).

#### 3.6.2 External validity

CanTox‐17 factor 4 (psychosis‐like experience/loss of reality) displays the highest correlation with the CAPE positive scale (*r* = 0.76; *p* ≤ 0.001). This indicates that they represent a common construct and supports construct validity of factor 4. Factor 4 correlates lower but significantly with the CAPE negative scale and the CAPE total score (both *r* = 0.27; *p* ≤ 0.001). CanTox‐17 factor 1 (anxiety and dysphoria) correlates at moderate to high levels with the CAPE negative scale (*r* = 0.43; *p* ≤ 0.001) and with the CAPE total score (*r* = 0.41; *p* ≤ 0.001). Also, it correlates with the CAPE depressiveness scale (*r* = 0.34; *p* ≤ 0.001). With CAPE positive, the correlation is at low to moderate levels (*r* = 0.27; *p* ≤ 0.001). CanTox‐17 factor 2 (confusion and disorientation) correlates significantly at intermediate levels with CAPE negative and CAPE total score (*r* = 0.25 and *r* = 0.21; both *p* ≤ 0.001), but not significantly with CAPE positive. The CanTox‐17 positive acute effects represented by factor 3 did not correlate significantly with any of the CAPE scales.

### 3.7 Correlations between CanTox‐17 scales and cannabis use patterns

Younger age at onset of cannabis use was significant correlated with higher levels of reported dysphoria and anxiety as acute cannabis intoxication states (CanTox‐17, scale 1), correlating with *r* = −0.233 (*p* = 0.003). No further associations were identified between patterns of cannabis use and intoxication effects.

## 4 DISCUSSION

CanTox‐17 is a reliable and valid but, due to the initial character of the study, preliminary German‐language instrument for the time‐efficient assessment of cannabis intoxication effects, based on the original English version (CEQ, Barkus et al., 2006). The four scales (paranoia/dysphoria, confusion/disorientation, euphoria/creativity, psychosis‐like/loss of reality) form both positive and negative acute intoxication effects. The decision for the final four‐factorial solution and against the five‐factorial solution was made after content inspection of the items. In particular, the items of factor 3 of the five‐factorial solution did not appear to be consistent in terms of content. In addition, the variance explanation of the final solution is higher and the external validity also proved to be more pronounced.

In contrast to the original CEQ of Barkus et al. (2006), CanTox‐17 focuses exclusively on acute intoxication effects. According to Quinn et al. (2017), we excluded questionnaire items that focus on subacute “after effects”. Thus, we circumvented potential problems with the questionable discriminatory power between “acute intoxication” and “subacute after effects,” which was discussed in the expert validation of our items. Accordingly, in a factor analysis by Birnbaum et al. (2017), the so‐called “after effect” items did not load on a separate factor but were distributed across all subscales.

Compared with the English‐language factor solutions, the two present scales “paranoia/dysphoria” and “euphoria/creativity” partly correspond with the factor solutions of the English colleagues (Barkus & Lewis, 2008; Birnbaum et al., 2017; Quinn et al., 2017), although there is some variation between the variants at the item level. The present scales 2 and 4 (“confusion/disorientation” and “psychosis‐like/loss of reality”) correspond most closely with the “distortions of reality and self‐perception” scale identified by Birnbaum et al. (2017), but also with Barkus and Lewis' (2008) “psychosis‐like effects,” although the latter were conceptualized as after‐effects.

Regarding external validity, CanTox‐17 scales 1, 2 and 4 correlate significantly with CAPE scales. So, it may be assumed that different facets of psychotic and depressive experiences are validly represented by the present instrument.

The option to operationalise both pleasant and different aversive acute effects of cannabis use with the CanTox‐17 intends for the first time in German‐speaking countries to analyse the significance of cannabis intoxication for different issues. Thus, cannabis‐induced psychosis‐like effects may be significant in the early detection of psychosis. After all, initial evidence exists that cannabis‐induced PLE occur particularly in individuals with vulnerability to psychosis (Takahashi et al., 2021). Corresponding acute effects could serve as a possible indicator of potentially increased psychosis risk, which would allow early preventive interventions. This is supported by the present association between younger age at first use of cannabis and the CanTox Scale 1 (paranoia/dysphoria), since early onset of use appears to be associated with increased risk for psychopathological development (Gage et al., 2016; Hamilton et al., 2017; Sideli et al., 2020). In addition, more in‐depth research on psychosis‐like acute effects could serve a better understanding of risk parameters for psychosis.

However, critics point out that PLE are not equivalent to full criteria of attenuated psychotic symptoms (APS) and brief intermittent psychotic symptoms (BIPS) as well defined risk markers of psychosis (Schultze‐Lutter et al., 2018). To psychotic‐like experiences on the other hand may overestimate the risk of psychosis: According to a large population study, only one out of 20 respondents with PLE met the criteria of APS/BIPS. These include course and frequency of their occurrence as important parameters for estimating psychosis risk, which are commonly not reported in epidemiological studies on PLE (Schultze‐Lutter et al., 2018).

Regarding manifest psychoses, cannabis induced PLE may be interesting based on the “discontinuation hypothesis”, postulating that some patients may reduce or stop cannabis use because of negative acute effects (Sami et al., 2019). Assuming a complex interaction between cannabinoids and neurophysiological vulnerability for psychosis (Hamilton, 2017), this would explain the relatively stable incidence of schizophrenia, although cannabis use now starts on average at an earlier age and tetrahydrocannabinol (THC) levels in cannabis have increased (Orth & Merkel, 2019). However, if increasing THC levels were associated with increased aversive acute effects, resultant reductions in use could make the steady incidence of psychoses comprehensible. Accordingly, high‐potency cannabis led to cessation of use due to aversive psychoactive effects in schizophrenia‐risk individuals who are particularly sensitive to THC effects (Sami et al., 2019). Cessation of use, in turn, was associated with fewer psychotic relapses and reduced treatment duration (Schoeler et al., 2016). However, research findings on the relationship between consumption effects and consumption behavior are inconsistent (Sami et al., 2020), and in the present study, cannabis use frequency did not appear to be associated with cannabis acute effects. In particular, acute substance effects appear to be less operant during prolonged use than during early stages of use (Fergusson et al., 2003; Le Strat et al., 2009; Sami et al., 2019), and the present sample has, after all, been using cannabis for an average of 7 years (difference between average age and age of first use). However, to the extent that acute cannabis effects may influence use behavior, at least in certain phases of use, this would have implications for the treatment of use disorders.

### 4.1 Limitations

The present data are based on retrospective estimates and are therefore prone to error. Furthermore, THC levels of cannabis consumed by subjects was not recorded, whereas Freeman et al. (2018) postulated an increased THC level in cannabis and Marconi et al. (2016) reported increased psychosis‐like intoxication effects at elevated THC levels. Differences in THC levels could thus lead to different factorial solutions of acute effects. Not to mention the unfortunately unrecorded possibility that synthetic cannabinoids were consumed, the ingredients of which are entirely unpredictable. The presence of psychiatric disease as an exclusion criterion was not recorded with standardized diagnostics and has only limited validity. In addition, data on subjective intoxication effects may be biased because subjects estimate a state in which they were acutely intoxicated, that is not in full possession of their mental capacities. And it is questionable whether acute intoxication is comparable in retrospective. In addition, to reduce bias in the data regarding the acute consumption effects, we limited the sample to acute cannabis users. However, the generalizability of the data might have been higher if former consumers had also been included in the study. Our concern was that a longer retrospective might lead to more recall bias. Moreover, the survey was conducted online. The prevailing pandemic corona situation did not allow for any other format. Thus, in the end, it cannot be ruled out that individual participants provided implausible information, which would have been more noticeable in a face‐to‐face situation. Moreover, recruitment in the online setting deviates from the otherwise usual recruitment criteria in scientific studies. It appears to be a viable alternative due to the lack of spatial barriers, but on the other hand, there are still no robust empirical data on possible biases in internet‐based samples. Data from correspondingly recruited samples may therefore be considered as non‐definitive exploratory work. This initially preliminary character of our findings is also based on a sample size that is rather small, although specific quality criteria are defined according to Hu and Bentler (1999). Follow‐up studies with a significantly larger sample should therefore replicate or optimise the presented factor structure of the CanTox‐17.

## 5 CONCLUSIONS

The sample size of 537 subjects enabled the cross‐validation of the CanTox‐17 by means of a random‐split. A 17 items version of the instrument is user‐friendly, as it can be performed in a time‐efficient manner. The four scales are also plausible in terms of content and have good properties in terms of model fit, reliability, and construct validity. Nevertheless, taking into account the limitations mentioned above, the result is to be understood as an initial impact that initiates research on acute cannabis effects in German‐speaking areas. And the presented factor structure is to be considered as a preliminary result until it has been sufficiently replicated or optimized by follow‐up studies. Such investigations should also compare patterns of acute intoxication effects in specific user groups and collect normative values for these groups (for example healthy vs. clinical samples, users with and without cannabis use disorder, past vs. current users, groups with different comorbidities, especially comorbid mental disorders with associated risk of psychosis such as schizotype or borderline disorder). Finally, follow‐up studies should analyse predictors associated with various acute intoxication effects. Further studies could focus on parameters predicted by intoxication effects (e.g., the issue of psychosis risk in cannabis‐induced psychosis‐like experiences).

However, following van Os and Guloksuz (2017), it should be cautioned against viewing any psychosis‐like intoxication state through a “schizo‐prism” and inferring inappropriate concepts of psychosis risk thereon. Analogous to the stricter APS and BIPS criteria relative to a lifetime occurrence of PLE, close attention should be paid to the point at which particular intoxication states are truly able to predict transition rates into clinically relevant psychosis. Which symptoms they are, how frequently they occur, and how long they should persist to justify a place on the psychosis continuum would be important questions for prospective follow‐up projects.

## AUTHOR CONTRIBUTIONS

Thomas Schnell contributed to conception and design of the study, coordinated the study and wrote the manuscript. Merle Schüler recruited data and performed statistical analyses. Steffen Moritz contributed to the interpretation of the results and revised the manuscript. All authors read and approved the final manuscript.

## CONFLICT OF INTEREST

The authors declare that they have no conflict of interest.

## Data Availability

The data that support the findings of this study are available from the corresponding author upon reasonable request.
